# Specific clinical and radiological characteristics of anti-NMDA receptor autoimmune encephalitis following herpes encephalitis

**DOI:** 10.1007/s00415-024-12615-7

**Published:** 2024-08-16

**Authors:** Pauline Dumez, Macarena Villagrán-García, Alexandre Bani-Sadr, Marie Benaiteau, Elise Peter, Antonio Farina, Géraldine Picard, Véronique Rogemond, Marie-Camille Ruitton-Allinieu, François Cotton, Mélodie Aubart, Marie Hully, Jean-Christophe Antoine, Bastien Joubert, Jérôme Honnorat

**Affiliations:** 1 French Reference Center on Paraneoplastic Neurological Syndromes and Autoimmune Encephalitis, Hôpital Neurologique Pierre Wertheimer, Hospices Civils de Lyon, 59 Bd Pinel, 69677 Bron cedex, France; 2 MeLiS-UCBL-CNRS UMR 5284, INSERM U1314, Université Claude Bernard Lyon 1, Lyon, France; 3 Department of Radiology, Hôpital Neurologique Pierre Wertheimer, Hospices Civils de Lyon, Bron, France; 4 Department of Radiology, Centre Hospitalier Lyon-Sud, Hospices Civils de Lyon, Pierre-Bénite, France; 5 CREATIS, INSERM U1044, CNRS UMR 5220, Université Claude Bernard Lyon 1, Villeurbanne, France; 6 Department of Neurology, Hôpital Universitaire de Saint-Etienne, Saint-Etienne, France; 7 Pediatric Neurology Department, Necker-Enfants-Malades Hospital, Assistance Publique-Hôpitaux de Paris, Paris, France

**Keywords:** Antibody-mediated encephalitis, *N*-methyl-d-aspartate receptor, Infectious encephalitis, Post-infectious

## Abstract

**Background:**

Herpes simplex virus encephalitis (HSE) frequently triggers secondary anti-*N*-methyl-d-aspartate receptor encephalitis (NMDARE), but markers predicting the occurrence of this entity (HSE-NMDARE) are lacking.

**Methods:**

We conducted a retrospective description of patients with HSE-NMDARE diagnosed between July 2014 and August 2022 and compared them to both patients with regular forms of HSE and NMDARE.

**Results:**

Among the 375 patients with NMDARE, 13 HSE-NMDARE were included. The median age was 19 years (0.5–73), 4/13 (31%) were children < 4 years old, and 7/13 (54%) were male. The median time between HSE and NMDARE onset was 30 days (21–46). During NMDARE, symptoms differed from HSE, including increased behavioral changes (92% vs 23%, *p* = 0.008), movements disorders (62% vs 0%, *p* = 0.013), and dysautonomia (54% vs 0%, *p* = 0.041). Compared to 21 patients with regular HSE, patients with HSE-NMDARE more often achieved severity-associated criteria on initial MRIs, with extensive lesions (11/11, 100% vs 10/21, 48%, *p* = 0.005) and bilateral diffusion-weighted imaging sequence abnormalities (9/10, 90% vs 6/21, 29%, *p* = 0.002). Compared to 198 patients with regular NMDARE, patients with HSE-NMDARE were more frequently males (7/13, 54% vs 43/198, 22%; *p* = 0.015) and children < 4 (4/13, 31% vs 14/198, 7%; *p* = 0.016), with a worse 12-month mRS (2[1–6] vs 1[0–6], *p* = 0.023).

**Conclusions:**

Herein, patients with HSE-NMDARE have a poorer long-term prognosis than patients with regular NMDARE. We report a greater rate of severity-associated criteria on initial MRIs for HSE-NMDARE compared to regular HSE, which may help identify patients with higher risk of HSE-NMDARE.

## Introduction

Herpes simplex virus encephalitis (HSE) is the most common infectious encephalitis worldwide with an incidence of 2–4 cases per million persons per year [1]. It is responsible for a high mortality, despite the appropriate use of intravenous acyclovir and the monitoring of severe patients in intensive care units [2], and for long-term sequelae in more than half of survivors [1, 3]. Notably, after initial improvement, 20–25% of patients subsequently harbor neurological deterioration compatible with autoimmune encephalitis (AE), associated with the detection of neuronal autoantibodies in the cerebrospinal fluid (CSF) and a worse functional status during follow-up [4, 5]. In two-third of patients, the autoantibodies found are directed against the *N*-methyl-d-aspartate receptor (NMDAR), while they target an unknown neuronal surface antigen in the remaining cases [4]. Of note, NMDAR encephalitis (NMDARE) is a mainly idiopathic entity in which symptoms are specific and usually responsive to immunotherapy; the disease mostly occurs in young women, and one-third of them carries ovarian teratomas [6, 7]. In patients with HSE-NMDARE, symptoms typically occur within 3 months after HSE onset and mimic those of the regular NMDARE clinical spectrum, including behavioral changes, psychiatric symptoms, seizures, and vigilance disorders [4, 8, 9]. Above all, their improvement after immunomodulatory treatments demonstrates the importance of rapidly distinguishing this entity from viral sequelae or relapse [10, 11].

Although HSE often triggers secondary NMDARE, there are few risk factors enabling physicians to predict HSE-NMDARE. To date, only the detection of autoantibodies in the CSF, and/or an increased blood type I interferon RNA signature are associated with a higher risk of HSE-NMDARE when performed at 21 days from HSE onset [4, 5]. However, since HSE is rare and HSE-NMDARE remains under-recognized, most patients do not benefit from state-of-the-art inflammatory monitoring, and parameters allowing to predict the disease remains needed.

In order to deepen knowledge and raise awareness regarding this entity, we described the clinical and radiological characteristics of patients with HSE-NMDARE diagnosed at the French Reference Center for Paraneoplastic Neurological Syndromes and Autoimmune Encephalitis (referred to below as the “French Reference Center”) and compared them both to patients with HSE not followed by AE, and to patients with NMDARE not preceded by HSE.

## Methods

### Patients

All patients with positive anti-NMDAR antibodies (NMDAR-Abs) tested in the French Reference Center from July 2014 to August 2022 and presenting with a clinical picture compatible to NMDARE [12] were retrospectively identified. NMDAR-Abs positivity was defined as the combination of CSF reactivity on indirect brain rodent immunohistofluorescence and positive cell-based assay on human embryonic kidney 293 cells expressing GluN1 (or NR1) and GluN2b (NR2b) subunits of the NMDAR antigen as previously described [13]. HSE was defined by a positive HSV-1 polymerase chain reaction (PCR) using CSF. Among identified patients, cases occurring within 12 months following an HSE diagnosis were described separately and are referred to as the HSE-NMDARE cohort. Their characteristics were compared to those of other patients with NMDARE for which appropriate clinical data were available, referred to as regular NMDARE. Data from magnetic resonance imaging (MRIs) of the HSE-NMDARE cohort at HSE diagnosis were compared to those of patients with HSE without secondary neurological worsening, diagnosed by a positive HSV-1 PCR using CSF and enrolled consecutively in a single-center (*Hospices Civils de Lyon*, France) during the same period [14]. These last were referred to as regular HSE after exclusion of patients who died within the first 3 weeks.

### Data collection

The clinical data of patients were retrospectively collected from medical records at HSE diagnosis, after acyclovir treatment, at NMDARE diagnosis, and at 3, 6, and, 12-month follow-ups from HSE onset. Their general characteristics were collected (age, sex, ethnicity, and medical history) and the main symptoms were categorized as follows: fever, seizures, vigilance disorders (decreased level of consciousness persisting for > 24 h), behavioral changes (comprising apathy, aggressiveness, or disinhibition), psychiatric disorders (comprising delirium, hallucinations, depression, or anxiety), movement disorders, speech and/or memory impairment, autonomic disturbances (comprising vasomotor/cardiac, pupillomotor, urogenital, secretomotor and gastrointestinal troubles) and sleep disorders (comprising insomnia or sleep–wake cycle disturbances). Memory and speech impairments were not assessed in patients younger than 4 years old. Disability was retrospectively measured with the modified Rankin scale (mRS) [15] at each time-point. A favorable outcome was defined by mRS ≤ 2 at 12-month follow-up.

Imaging and laboratory findings were collected at each time-point when performed. HSE radiological severity criteria were defined as either extensive lesions reaching more than three lobes on fluid-attenuated inversion recovery (FLAIR) sequence or bilateral abnormalities on diffusion-weighted imaging (DWI) sequence [16, 17]. During NMDARE, enlargement of lesions was defined as an abnormal signal in a newly involved cerebral lobe on T2 sequence. Available MRIs from the HSE-NMDARE cohort and patients with regular HSE were analyzed both by a neurologist (PD) and a neuroradiologist (ABS) blinded to the autoimmune status. MRIs of patients with regular NMDARE were classified using radiological reports.

### Statistical analysis

All analyses were performed using R statistical software (version 2023.06.2 + 561, Vienna, Austria). Data are expressed as count (percentage) or median (range). In the HSE-NMDARE cohort, paired clinical and paraclinical data during both the HSE and NMDARE post-HSE phases were compared using the McNemar test for categorical variables, or the Wilcoxon rank test for continuous variables. Characteristics from the HSE-NMDARE cohort were compared with those of patients with regular NMDARE and regular HSE using the Chi^2^ or Fisher’s exact test for categorical variables, the Mann–Whitney *U* test for continuous variables, and the Kruskal–Wallis for ordinal variables, as appropriate. All statistical tests were two-sided and a *P* value < 0.05 was considered significant.

### Ethical approval

The included patients or their legal representatives received written information, and non-opposition was obtained by the referent physician from all the patients. This study was approved by the institutional review board of the *Hospices Civils de Lyon* and registered in ClinicalTrials.gov (N°20-87). The *Commission nationale de l’informatique et des libertés* (CNIL) also approved the study for data collection (No. 20-112) according to the Act n°78-17 of January 6, 1978 on data processing, data files, and individual liberties.

## Results

### Characteristics of the patients

**Fig. 1:**
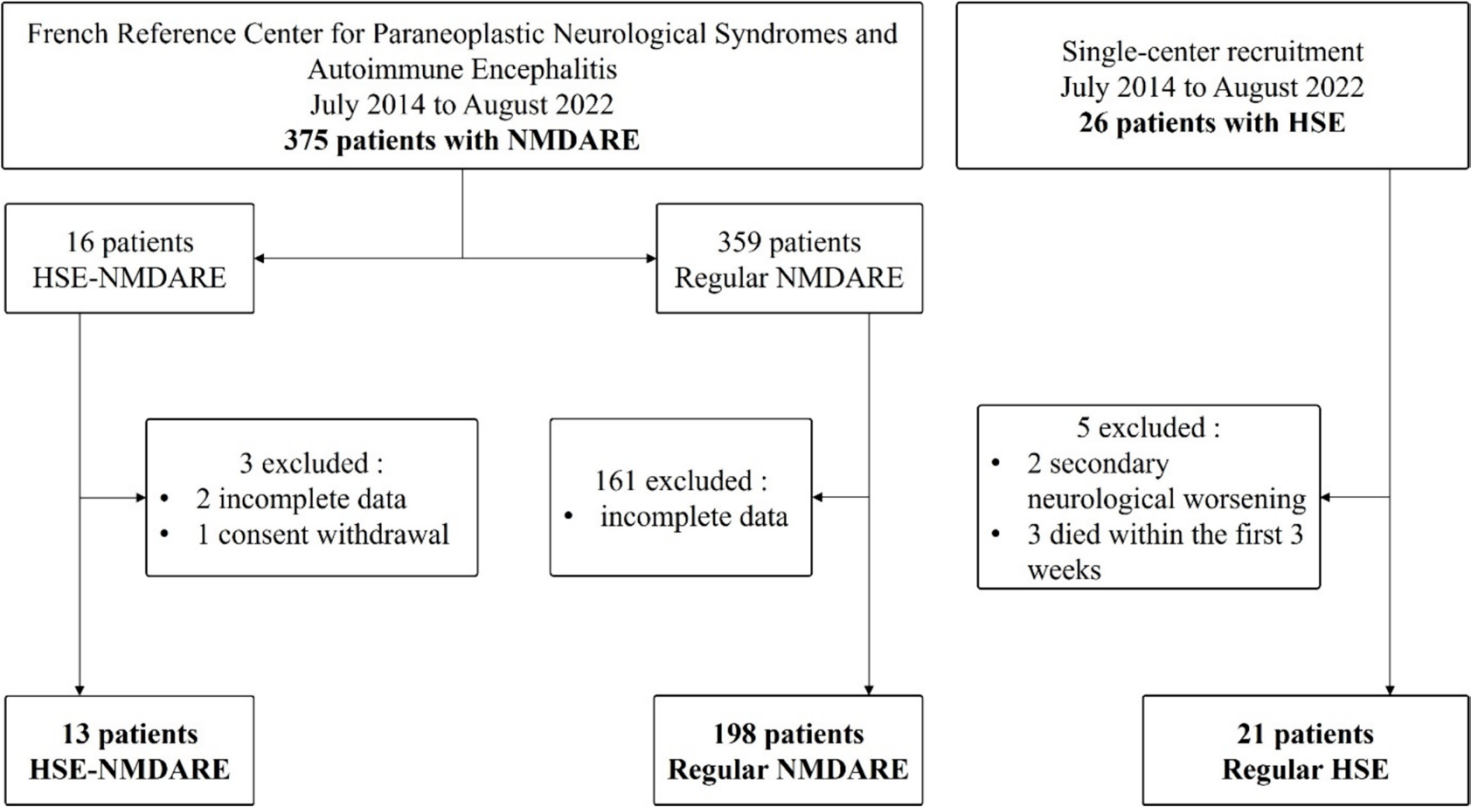
Study flowchart. Diagram of patients’ inclusion. Abbreviations: *HSE* herpetic encephalitis, *NMDARE* anti-*N*-methyl-d-aspartate receptor encephalitis, *MRI* magnetic resonance imaging

Among the 375 patients diagnosed with NMDARE in the Reference Center, 16 (4.3%) met the criteria for HSE-NMDARE; 3 were excluded, 2 due to missing clinical data and 1 after consent withdrawal. Finally, 13 patients with HSE-NMDARE were included in the analysis (Fig. 1). The median age was 19 years (range 0.5–73), 4 (31%) were children younger than 4 years old. Seven (54%) were male, 10 (77%) were Caucasian, and 1 patient was pregnant and has already been reported elsewhere [18]. Two patients (15%) had a history of severe herpetic infection (one patient had a previous first episode of HSE, and one had ocular herpetic lesions), 3 (23%) had a personal history of autoimmunity (one patient had psoriasis, one had allergic asthma, and one had systemic sarcoidosis).

### Clinical presentation of patients with HSE-NMDARE

At HSE diagnosis, 13/13 (100%) patients had fever, 9/9 (100%) had memory impairment, 9/13 (69%) had seizures, 8/13 (62%) had vigilance disorders, 5/10 (50%) had speech impairment, 6/13 (46%) had headaches, 3/13 (23%) had behavioral changes, 3/13 (23%) had motor dysfunction and 1/13 (8%) had psychiatric symptoms. The median mRS score at HSE diagnosis was 5 (range 4–5). All patients received acyclovir for 21 days with a median time from HSE onset of 5 days (range 0–10), and 4/13 (31%) patients received early corticosteroid therapy with various dosages. After acyclovir treatment and before NMDARE onset, the median mRS score decreased to 2 (range 0–5), two patients fully recovered, two had no improvement, while nine experienced partial recovery of symptoms.

During NMDARE, all patients developed new neurological symptoms comprising behavioral changes (12/13, 92%), memory impairment (8/9, 89%), vigilance disorders (8/13, 62%), seizures (8/13, 62%), movement disorders (8/13, 62%), speech impairment (5/9, 56%), dysautonomia (7/13, 54%), psychiatric disorders (6/13, 46%), motor dysfunction (5/13, 38%), fever (4/13, 31%), sleep disorders (4/13, 31%), and ataxia (3/13, 23%). Movement disorders comprised dyskinesia (7/8, 88%), choreoathetosis (2/8, 25%), or dystonic postures (2/8, 25%), and involved the orofacial region (8/8, 100%) and/or the limbs (5/8, 63%). The median time between HSE and NMDARE onset was 30 days (range 21–46), and the median time between NMDARE onset and the positive antibody testing was 17 days (range 7–74). In 2/13 patients (15%), new-onset NMDARE symptoms were masked by those of HSE and recognized later due to absence of improvement after acyclovir. During NMDARE, the median mRS score peaked at 5 (range 4–5), a total of 6/13 (46%) patients were hospitalized in intensive care unit (ICU), and the median length of stay was 56 days (range 12–85). At that time, all patients had a negative HSV-1 PCR in the CSF, and oligoclonal bands were positive in 5/5 patients (100%). Before NMDARE diagnosis was confirmed, 5/13 (38%) patients received a second course of acyclovir. Then, all patients received immunotherapy after a median time of 14 days (range 4–36) from NMDARE onset. As first-line therapy, all patients received intravenous immunoglobulins, 9/13 (69%) received high doses of intravenous corticosteroid, and 2/13 (15%) plasma exchanges. As second-line therapy, 9/13 (69%) received rituximab, 3/13 (23%) cyclophosphamide, 2/13 (15%) mycophenolate mofetil, and 1/13 (8%) patient intrathecal methotrexate. Cancer screening using thoracic computed tomography scan and/or pelvic ultrasound was negative in all adult patients with NMDARE post-HSE, the search for malignancy was not systematically performed in children.

The median follow-up in HSE-NMDARE patients was 12 months (range 9–60). At 3 months of follow-up, the median mRS was 4 (range 1–6), 1 patient died from a respiratory tract infection due to his neurological condition. At 12 months, 1 patient was lost to follow-up; the median mRS decreased to 2 (range 1–4), 7/12 patients (58%) had a favorable outcome, but none of them ever achieved mRS = 0. Sequelae documented at last follow-up were seizures requiring antiepileptic treatment in 12/13 patients (92%), memory impairment in 10/13 (77%), behavioral disorders in 8/13 (62%), and movements disorders in 2/13 (15%). No patient presented infectious, nor autoimmune relapse. Persistent anti-NMDAR antibodies were found in the CSF of 4/7 patients (57%) at 3 months of follow-up, 3/6 (50%) at 6 months, and 1/5 (20%) at 12 months. Clinical characteristics, treatments, and outcomes are reported in Table 1.

**Table 1:** Clinical features, treatments, and outcomes of patients with HSE-NMDARE

N^o^, age, sex	NMDARE onset: time after HSE, symptoms	Peak mRS NMDARE	Immunotherapy	12-month mRS
N^o^1, 48, M	**Day 32.** Fever, behavior trouble (agitation), confusion, seizure, dyskinesia (orofacial), dysautonomia, vigilance disorders, ataxia	5	IvMP, IvIg, rituximab	Death at 3 months
N^o^2, 52, F	**Day 46.** Severe aphasia, depression, dyskinesia (orofacial), urinary retention, apraxia	5	IvMP, IvIg, cyclophosphamide, mycophenolate mofetil	2
N^o^3, 6 mo, M	**Day 26.** Behavior trouble (agitation), hypotonia, focal seizures	4	IvMP	1
N^o^4, 13, M	**Day 35.** Behavior trouble (agitation), anxiety, aggressiveness, depression	4	IvIg, rituximab	1
N^o^5, 11, F	**Unknown.** Fever, alertness disorder, behavior trouble (auto aggressive), psychiatric symptoms	5	IvIg	4
N^o^6, 49, M	**Day 24.** Dystonia (larynx and diaphragm), memory disorder, central ventilatory disorder, vigilance disorders, tremor	4	IvIg	2
N^o^7, 1, M	**Day 24.** Choreoathetosis, dyskinesia (orofacial), behavior trouble (agitation), seizures, swallowing disorders, hypotonia	5	IvMP, IvIg, rituximab	1
N^o^8, 30, F	**Day 33.** Confusion, delirium, memory disorder, alertness disorder, polydipsia, dyskinesia (orofacial), ataxia, seizures, limb paresis, urinary retention, apraxia	5	IvMP, IvIg, rituximab, cyclophosphamide	3
N^o^9, 3, F	**Day 25.** Fever, vigilance disorders, behavior trouble (agitation), dystonia (limbs and face), dyskinesia (limbs), hypotonia, hemiparesis, choreoathetosis, swallowing disorders, dysautonomia	5	IvMP, IvIg, PLEX, rituximab, ITh methotrexate	Unknown
N^o^10, 73, M	**Day 43.** Fever, vigilance disorders, swallowing disorders, memory disorders, hypotonia	5	IvMP, IvIg, rituximab	3
N^o^11, 10 mo, F	**Day 27.** Fever, dyskinesia (facial, limbs), sleep troubles (insomnia), hemiparesis, seizures, spasms	5	IvMP, IvIg, rituximab	4
N^o^12, 51, H	**Day 45.** Behavior trouble, seizures, confusion, depression, memory disorder	5	IvMP, IvIg, PLEX, rituximab	2
N^o^13, 19, F	**Day 21.** Behavior trouble (agitation), psychiatric symptoms (delirium, anxiety, depression), dysautonomia, confusion, memory disorder, aphasia	5	IvIg, cyclophosphamide, rituximab	1

Abbreviations: *M* male, *F* female, *HSE* herpetic encephalitis, *NMDARE* anti-*N*-methyl-d-aspartate receptor encephalitis, *mRS* modified rankin score, *IvMP* intravenous methylprednisolone, *IvIg* intravenous immunoglobulins, *PLEX* plasma exchanges, *Mo* months, *ITh* intrathecal

**Fig. 2:**
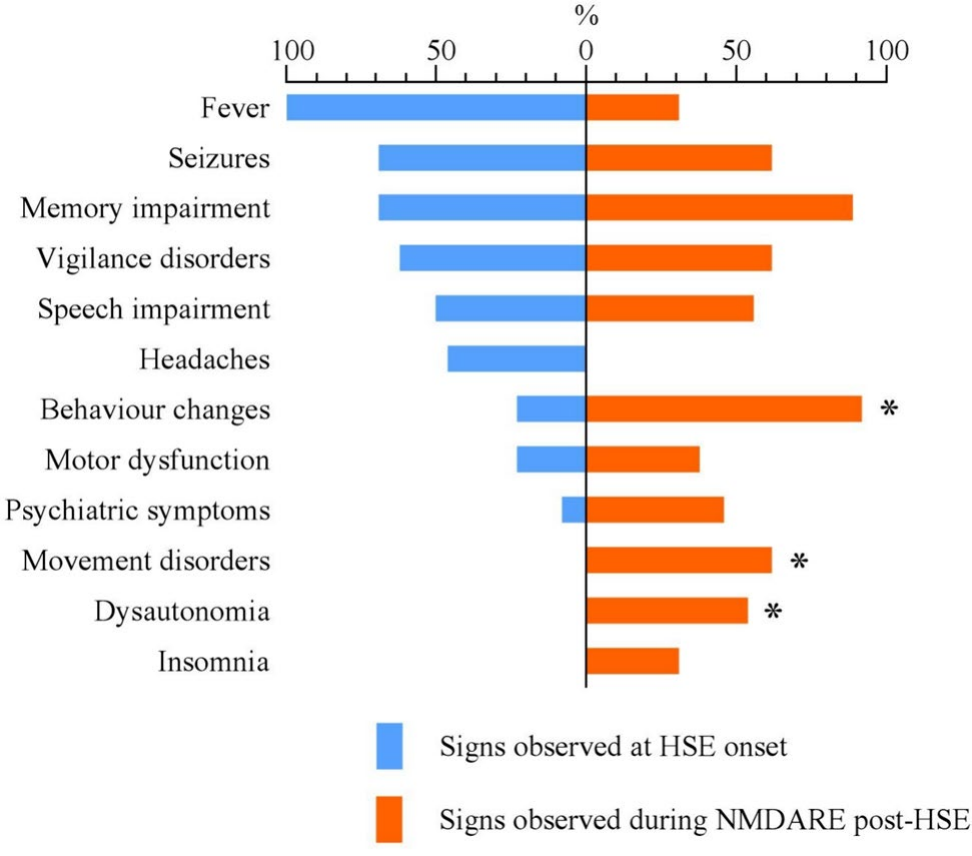
Variation of symptoms between HSE onset and NMDARE post-HSE, (%, *n* = 13)*.* Comparison of symptoms at HSE onset and during NMDARE post-HSE in the HSE-NMDARE cohort, frequencies are represented as percentages. For the same 13 patients, HSE symptoms are represented on the negative axis in blue, NMDARE symptoms on the positive axis in orange. Speech and memory impairment were only assessed for patients aged ≥ 4 years (*n* = 9). *P* values < 0.05 are indicated with *. Abbreviations: *HSE* herpetic encephalitis, *NMDARE* anti-*N*-methyl-d-aspartate receptor encephalitis

When comparing the symptoms observed during NMDARE post-HSE with those occurring in the HSE phase, behavioral changes (92% versus 23%, *p* = 0.008), movements disorders (62% vs 0%, *p* = 0.013), and dysautonomia (54% vs 0%, *p* = 0.041) were more frequent (Fig. 2). The median white blood cells (WBC) count was lower during NMDARE than at HSE onset (22 WBC/mm^3^ [range 2–192] vs 150 [range 80–520], *p* = 0.019), with a downward trend regarding protein levels (0.78 g/L [range 0.28–1.6] vs 1.04 [range 0.22–1.97], *p* = 0.697).

The cohort size did not allow statistical comparison according to patient’s age, however, there was a trend toward a shorter median time between HSE and NMDARE onset in children aged < 4 compared to older patients (25.5 days [range 24–27] vs 34 [range 21–46]). Choreoathetosis was found only in children < 4 years of age (2/3, 75% vs 0/5), whereas psychiatric symptoms seemed specific to older patients (6/9, 67% vs 0/4). There was also a trend in patients < 4 years of age to more frequently present with vigilance disorders (7/9, 78% vs 1/4, 25%). The proportion of fever (2/4, 50% vs 2/9, 22%), seizures (3/4, 75% vs 5/9, 56%), confusion (3/4, 75% vs 9/9, 100%), motor disorders (2/4, 50% vs 3/9, 33%) and movements disorders of any kind (3/4, 75% vs 5/9, 56%) seemed similar.

### Brain magnetic resonance imaging findings in HSE-NMDARE and comparison with regular HSE

**Fig. 3:**
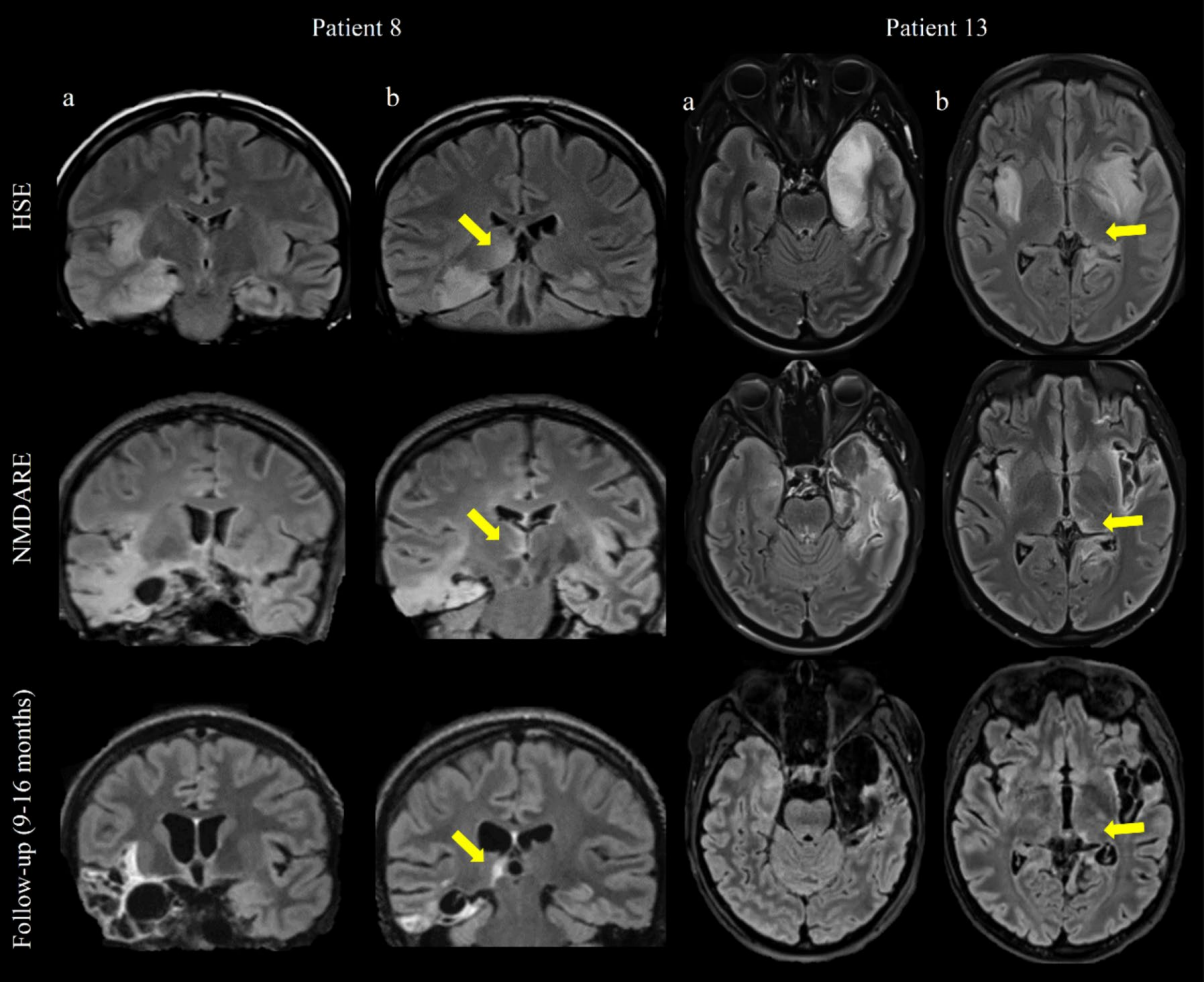
Temporal and thalamic patterns on longitudinal MRIs of patients with HSE-NMDARE*.* T2-Fluid-attenuated inversion recovery sequences are shown for patients 8 and 13. For each patient, the temporal pattern is shown on the left (**a**), and the thalamic on the right with yellow arrows (**b**). Longitudinal MRIs are shown at HSE diagnosis (first line), NMDARE diagnosis (second line), and follow-up (third line). Abbreviations: *HSE* herpetic encephalitis, *NMDARE* anti-*N*-methyl-d-aspartate receptor encephalitis

In 11 HSE-NMDARE patients, MRIs were available in both the HSE and NMDARE post-HSE phases, and all (22/22) were abnormal. During HSE phase, 11/11 (100%) patients had bilateral lesions involving more than 3 lobes in FLAIR or T2 sequence; the temporal lobe was involved in all patients, the insula in 10/11 (91%), the frontal lobe in 9/11 (82%), the parietal lobe in 4/11 (36%), and the occipital lobe in 4/11 (36%). At HSE diagnosis, DWI signal abnormalities were systematically observed, being bilateral in 9/10 patients (90%, not interpretable in 1 patient due to metal braces). In addition, a parenchymal enhancement in post-contrast 3D T1-weighted MRI was observed in 2/9 (22%) patients and hemorrhagic suffusions in T2*-weighted imaging were observed in 3/10 (30%). At NMDARE diagnosis, enlargement of previous hyperintense FLAIR/T2 sequence lesions was observed in 8/11 patients (73%), a post-contrast parenchymal enhancement was observed in 5/9 patients (56%), and a severe atrophy was found in 8/11 patients (73%). Eight patients (54%) had available follow-up MRIs performed between 2 and 16 months from the NMDARE diagnosis, all revealed severe and worsening temporo-mesial atrophy (Fig. 3).

**Fig. 4:**
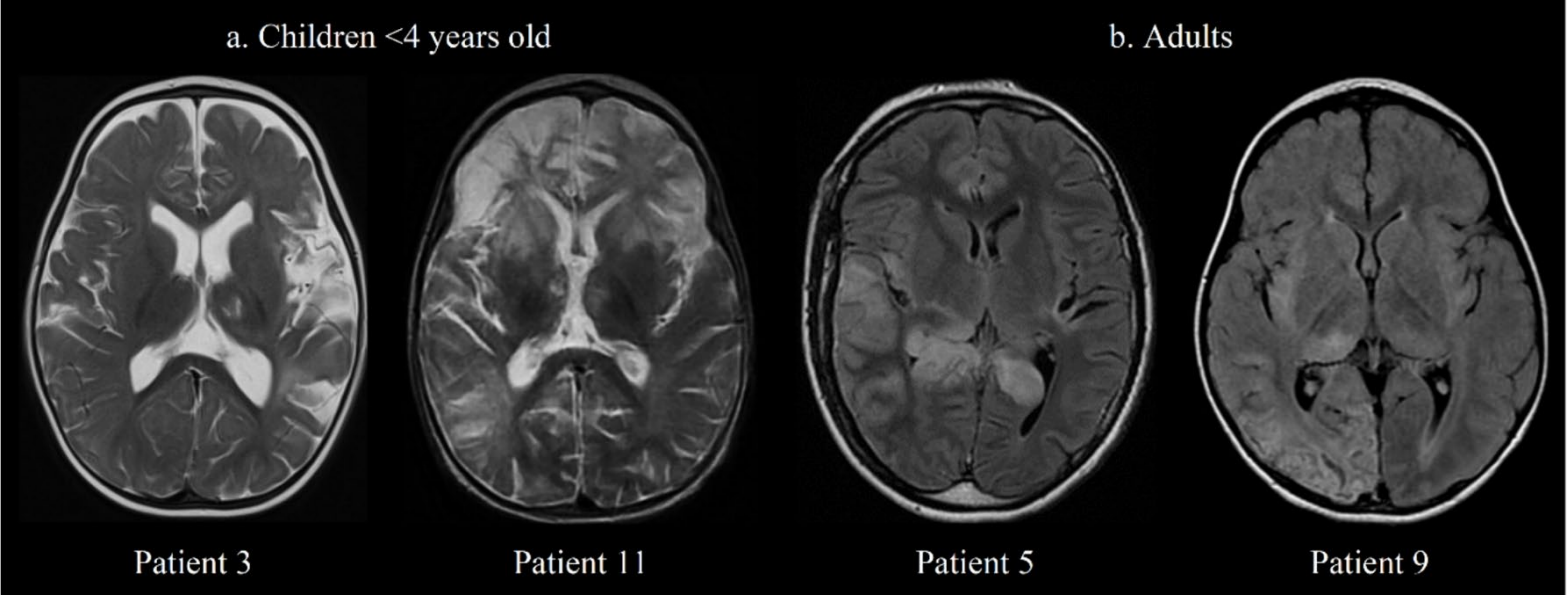
Topography of thalamic lesions in patients with HSE-NMDARE. The characteristics of thalamic lesions are shown for 4 patients at NMDARE diagnosis, according to their age. On the left (**a**), T2 sequence shows median/anterior thalamic lesions in patients 3 and 11, who were children < 4 years old. On the right (**b**), FLAIR sequence shows hyperintensity in the pulvinar region in adult patients 5 and 9. Abbreviations: *FLAIR* T2-fluid-attenuated inversion recovery, *NMDARE* anti-*N*-methyl-d-aspartate receptor encephalitis

At HSE diagnosis, thalamic lesions were observed in 8/11 patients (73%) in FLAIR/T2 sequence and in 2/10 (20%) in DWI sequence. At NMDARE diagnosis, they were observed in all patients (11/11) and remained systematically visible on follow-up imaging (8/8) with apparent nuclear atrophy. Thalamic lesions were ipsilateral to the most HSE-damaged hemisphere, they reached the dorsomedial and pulvinar regions in 9/11 patients (Fig. 4). In two patients, both < 4 years old, thalamic lesions were more anterior and bilateral. Their frequency, among all sequences, was similar in patients who reported seizures during HSE and those who did not (6/8, 75% vs 2/3, 67%).

Available MRIs of patients with HSE-NMDARE at HSE diagnosis (11/13) were compared to those from 21 patients with regular HSE who did not ensure secondary clinical worsening (Fig. 1). Regarding the general characteristics of the patients, the median age was lower in the HSE-NMDARE group (13 years old [range 1–73] vs 55 [range 1–82], p = 0.009), but the female proportion (6/11, 55% vs 9/21, 43%, *p* = 0.500), the median mRS at HSE diagnosis (5 [range 4–5] vs 5 [range 4–5], *p* = 0.699), as well as the time from HSE onset to MRI (4 days [range 2–13] vs 4 [range 0–13], *p* = 0.700) were similar. Patients with HSE-NMDARE more often achieved radiological severity criteria at HSE diagnosis, both on extensive lesions > 3 lobes (11/11, 100% vs 10/21, 48%, *p* = 0.005) and bilateral DWI abnormalities (9/10, 90% vs 6/21, 29%, *p* = 0.002). There was a trend toward more frequent FLAIR thalamic lesions in patients with HSE-NMDARE (8/11, 73% vs 7/21, 33%, *p* = 0.063), which was not observed in DWI sequence (2/10, 20% vs 4/21, 19%, *p* > 0.999). The frequency of parenchymal enhancement in post-contrast 3D T1-weighted MRI (2/9, 22% vs 5/17, 29%, *p* > 0.999) or bleeding in T2*-weighted imaging (3/10, 30% vs 4/18, 22%, *p* = 0.673) were similar in both groups.

### Comparison of HSE-NMDARE with regular NMDARE

Compared to 198 patients with regular NMDARE, patients with HSE-NMDARE were more frequently males and children < 4 (Table 2), had more frequently abnormal brain MRIs, and greater median protein levels in the CSF. The frequency of the main symptoms associated with NMDARE was similar in both groups. The rate of abnormal EEGs, the median WBC count in the CSF, the presence of an intrathecal immunoglobulin secretion (oligoclonal bands) and the positivity of NMDAR-Abs in the serum were also equivalent. Therapeutic management did not differ regarding the type and the total amount of immunotherapy received; however, patients with HSE-NMDARE had a higher median mRS at 3 months and 12 months of follow-up.

**Table 2:** Comparison of HSE-NMDARE with regular NMDARE

	HSE-NMDARE *n* = 13	Regular NMDARE *n* = 198	*p* value
General			
Males, *n* (%)	7 (54)	43 (22)	0.015
Age, median (range)	19 (1–73)	21 (2–80)	NS
Children < 4 years old, *n* (%)	4 (31)	14 (7)	0.016
Caucasian, *n* (%)	10 (77%)	91/149 (61)	NS
Symptoms			
Behavior changes, *n* (%)	12 (92)	187/197 (95)	NS
Memory impairment, *n* (%)	8/9 (89)	164/180 (91)	NS
Seizures, *n* (%)	8 (62)	151/192 (79)	NS
Vigilance disorders, *n* (%)	8 (62)	109/192 (57)	NS
Movement disorders, *n* (%)	8 (62)	125/194 (64)	NS
Dysautonomia, *n* (%)	7 (54)	82/191 (43)	NS
Sleep disorders, *n* (%)	4 (31)	68/190 (36)	NS
Speech impairment, *n* (%)	5/9 (56)	111/177 (63)	NS
Explorations			
Abnormal MRI, *n* (%)	13 (100)	62/177 (35)	< 0.001
Abnormal EEG, *n* (%)	12 (92)	152/182 (84)	NS
CSF WBC count/mm^3^, median (range)	18 (2–192)	22 (0–290)	NS
CSF proteinorachia g/L, median (range)	0.79 (0.28–1.74)	0.40 (0.10–2.50)	< 0.001
Oligoclonal bands, *n* (%)	5/5 (100)	37/52 (71)	NS
NMDAR-Abs in serum, *n* (%)	5/7 (71)	18/24 (75)	NS
Treatment			
Methylprednisolone iv., *n* (%)	9 (69)	157/183 (86)	NS
Immunoglobulins iv., *n* (%)	13 (100)	174/183 (86)	NS
Plasma exchanges, *n* (%)	2 (15)	46/188 (24)	NS
Rituximab, *n* (%)	9 (69)	141/173 (82)	NS
Total of molecules, median (range)	3 (1–6)	3 (1–8)	NS
Evolution			
Maximal mRS, median (range)	5 (3–6)	5 (1–5)	NS
3 months mRS, median (range)	4 (1–6)	3 (0–6)	0.046
12 months mRS, median (range)	2 (1–6)	1 (0–6)	0.023

Abbreviations: *HSE* herpetic encephalitis, *NMDARE* anti-*N*-methyl-d-aspartate receptor encephalitis, *MRI* magnetic resonance imaging, *EEG* electroencephalogram, *CSF* cerebrospinal fluid, *WBC* white blood cell, *Abs* antibodies, *Iv* intravenous, *mRS* modified Rankin Score, *NS* non-significant

## Discussion

In the present study, we described a cohort of 13 patients with HSE-NMDARE from a clinical and radiological perspective. Clinically, we observed a biphasic disease in which new symptoms of encephalitis occurred mostly within 2 months after HSE onset, in line with previous literature [4, 9, 19]. On the radiological aspect, we reported a high rate of severity criteria at HSE onset in patients subsequently developing NMDARE as well as a frequent thalamic involvement.

In accordance with previous studies, we reported a median time between HSE and AE of approximately 1 month [4, 9]. Although some symptoms were masked by HSE sequelae at relapse, the present cohort confirmed that new specific symptoms from the NMDARE spectrum such as behavioral changes, movement disorders, and dysautonomia were discriminant and made the diagnosis recognizable. In line with a review of 43 cases reported in the literature and two prospective studies, none of the patients herein who received immunotherapy during NMDARE developed a new viral replication after a median follow-up of 12 months [4, 5, 9].

We provided a comparison between HSE-NMDARE and regular NMDARE from the same large patient cohort, and extended previous results from a smaller pediatric cohort [19]. We found younger patients in the HSE-NMDARE group as well as a balanced sex ratio instead of the young adult female predominance observed in regular NMDARE, and hypothesize that this might reflect the preferential HSE distribution in extreme ages [20]. In addition, we report very similar clinical features in patients with regular NMDARE and HSE-NMDARE at the time of AE, which provide additional weight to their shared autoimmune origin. Despite immunotherapy conducted according to the same references, patients with HSE-NMDARE had a drastically worse 12-month prognosis than regular NMDARE, and none of them fully recovered. It is very likely that this difference is explained by irreversible loss of brain parenchyma due to viral-induced necrosis [14].

In the present HSE-NMDARE cohort, we made several interesting radiological observations. First, we systematically observed severity-associated criteria at HSE onset, comprising extensive lesions (> 3 lobes) and/or bilateral DWI abnormalities, whereas both of these features are reported in only one-third of HSE MRIs [16, 17]. Conversely, previous studies did not find any difference regarding the volume of damaged brain due to HSE on FLAIR and DWI sequences between patients who further developed AE and those who did not. Therefore, it could be hypothesized that stratification using validated criteria may have revealed this radiological difference [4]. Through comparison to a group of patients with regular HSE, we confirmed this observation since these severity criteria were two times more frequent in the HSE-NDMARE cohort. Second, we observed frequent thalamic lesions on MRIs of patients with HSE-NMDARE, i.e., in 2/3 of them at the time of HSE, and in 100% after NMDARE diagnosis, whereas they were encountered twice less frequently in patients with regular HSE as well as in the literature [16]. The signification of this finding is unclear since thalamic lesions remain underexplored beyond the acute phase of HSE and, to our knowledge, has not been previously investigated in HSE-NMDARE. Interestingly, left thalamic lesions are strongly associated with a poor prognosis when visible on DWI at HSE onset [16], which could be related to a peri- or post-ictal context such as the known “pulvinar sign” [21, 22]. Of note, we did not find any association between thalamic abnormalities on MRI and reported seizures. Furthermore, both the extension to several thalamic nuclei and the irreversibility of lesions on follow-up MRIs observed were surprising in this context. An alternative hypothesis involves the thalamocortical diaschisis, defined as projections from specific cortical structures to thalamic *nuclei* [23–25], and are suggestive of deafferentation or viral spread mechanisms. Although it is premature to assert the association of these radiological lesions with autoimmunity, Japanese encephalitis, the second largest cause of post-infectious NMDARE, has also a preferred tropism for diencephalon with frequent thalamic involvement on MRIs [26]. Therefore, we speculate that the radiological severity of HSE lesions, and potentially their extension to thalami, may be involved in the pathogenesis of a subsequent AE.

The present study has several limitations, including its retrospective nature and the small number of included patients in the HSE-NMDARE group. Nevertheless, the use of strict selection criteria (including positivity of the CSF regarding HSV-1 PCR at HSE onset and of anti-NMDAR IgG at NMDARE diagnosis) warrants high diagnostic accuracy herein. Moreover, the context of the French Reference Center allowed us to provide the long-term outcomes of these patients, with a median follow-up of 12 months since HSE onset, and to perform a reliable comparison between HSE-NMDARE and regular NMDARE. With the aim to focus on the HSE-NMDARE entity, in which the pathogenicity of antibodies found after HSE seems equal to that of regular NMDARE [27], we did not study patients with AE following HSE harboring other neuronal surface autoantibodies. Finally, we acknowledge that, since the expected prevalence is approximately 20% of that of HSE, it is likely that HSE-NMDARE is still under-diagnosed in France. In order to improve this issue, we recommend a close neurological follow-up in all HSE patients. A new CSF exploration with the search for neuronal autoantibodies - and especially anti-NMDAR - is recommended for patients with clinical worsening.

In conclusion, patients with HSE-NMDARE have a poorer long-term prognosis than patients with regular NMDARE, reflecting the irreversible neuronal loss caused by the viral infection. MRIs of these patients reveal greater rates of HSE severity-associated criteria when compared to patients with regular HSE, which suggests that baseline MRI features may help identifying patients with higher risk of HSE-NMDARE. Further studies confirming this hypothesis are warranted since the identification of such patients could reduce diagnostics and therapeutics delay through strengthened neurological monitoring.

## Data Availability

Additional data may be shared by the corresponding author on reasonable request, maintaining anonymisation of individual patient data.
